# Gender differences in the relationship between obsessive-compulsive symptoms and suicide risk among psychiatric outpatient adolescents: the role of depressive symptoms, anxiety symptoms, and sleep disturbances

**DOI:** 10.3389/fpubh.2025.1553066

**Published:** 2025-04-23

**Authors:** Xinwei Fang, Xiulian Qian, Xinzhu Hu, Huilan Jiang, Weiming Hu

**Affiliations:** Department of Child and Adolescent Psychiatry, The Third Hospital of Quzhou, Quzhou, Zhejiang, China

**Keywords:** obsessive-compulsive, suicide risk, depressive, anxiety, sleep disturbances, adolescents, outpatient

## Abstract

**Background:**

Suicide has become one of the leading causes of death among adolescents, with an increased risk observed in the psychiatric outpatient population. Therefore, exploring its risk factors is crucial. Obsessive-compulsive symptoms, being common in this patient group, warrant investigation into their impact mechanisms on suicide risk.

**Methods:**

This study enrolled 526 outpatient adolescents [396 females (75.29%); Mage = 15.39, SD = 1.23] who completed relevant questionnaires and provided demographic data during their clinic visit.

**Results:**

Obsessive-compulsive symptoms positively predicted suicide risk in both males and females, with depressive symptoms mediating this effect. Sleep disturbances played a mediating role only in females, while anxiety symptoms did not mediate the relationship in either gender.

**Conclusion:**

Clinicians should pay closer attention to adolescents presenting with obsessive-compulsive and depressive symptoms, as well as female adolescents with sleep disturbances, to mitigate their elevated suicide risk.

## Introduction

### Suicide risk in adolescents

Suicide is a global public health issue, and among adolescents aged 15–19 worldwide, it is the second leading cause of death for females and the third leading cause for males (1). In adolescents attending psychiatric outpatient clinics, the risk of suicide is significantly higher than in the general population (2). Therefore, identifying the factors influencing the risk of suicide among adolescents attending psychiatric outpatient clinics is of great significance for suicide prevention. Researchers have proposed a series of theories regarding suicidal behavior and suicide risk. Among these, the Integrated Motivational-Volitional (IMV) model of suicidal behavior provides a more comprehensive explanation of the factors influencing suicide risk and the process from suicide risk to suicidal behavior (3).

### Obsessive-compulsive symptoms and suicide risk

The IMV model divides the emergence of suicidal behavior into the pre- motivational phase, the motivational phase, and the volitional phase. The motivational phase is a critical phase in the formation of suicide risk, where feelings of entrapment and defeat during the motivational phase are significant precursors to the development of suicide risk. When individuals fail to control obsessive thoughts or behaviors, they are likely to experience a sense of defeat, leading to the emergence of suicide risk.

Obsessive-compulsive symptoms primarily consist of persistent and intrusive thoughts that provoke anxiety (obsessions), as well as repetitive behaviors performed to control these intrusive thoughts (compulsions) (4). In many cases, obsessive-compulsive symptoms begin in childhood and significantly impact the patient’s quality of life, well-being, and overall functioning (5). Consequently, obsessive-compulsive symptoms are likely to contribute to suicide risk. A meta-analysis involving 61 studies showed that obsessive-compulsive symptoms are associated with a higher risk of suicide; one in ten patients with obsessive-compulsive disorder attempt suicide in their lifetime, one-third currently have suicide risk, and approximately half have had suicide risk at some point (6). Although numerous studies have illustrated the relationship between obsessive-compulsive symptoms and suicide risk, the underlying mechanisms remain unclear. Moreover, both suicide risk and obsessive-compulsive symptoms exhibit gender differences (7–9), hence this study aims to investigate the mechanisms by which obsessive-compulsive symptoms influence suicide risk, as well as the gender differences involved.

### Role of depressive symptoms

In the IMV model, feelings of entrapment are a precursor to suicide risk, and depressive symptoms, as one of the consequences of entrapment, may also play a unique role (10). The role of depression in suicide risk has been demonstrated in numerous studies (11–14). Additionally, depressive symptoms as a common secondary symptom of obsessive-compulsive symptoms have been extensively validated in children (15) and particularly in adolescents (16, 17).When depressive symptoms are secondary to obsessive-compulsive symptoms, they may lead to more severe impairment (18) and a higher risk of suicide (19). Longitudinal studies show that obsessive-compulsive symptoms can predict depressive symptoms over a two-year follow-up (20), with more severe obsessive-compulsive symptoms indicating more severe depressive symptoms in the following year (21). In summary, depressive symptoms may mediate the relationship between obsessive-compulsive symptoms and suicide risk.

### Role of anxiety symptoms

Anxiety symptoms, as a common psychological issue among adolescents attending psychiatric outpatient clinics (22), may also contribute to suicide risk (23). In the IMV model, thoughts about the future are one of the significant predictors of suicide risk. Moreover, one of the main characteristics of anxiety symptoms is pessimism and worry about the future (24). Additionally, avoidance behavior, as one of the core symptoms of anxiety, may lead to social isolation, thwarted belongingness, and loneliness, which are critical risk factors for suicide (3, 25). A meta-analysis involving 309,974 patients with anxiety disorders and related conditions indicates that compared to those without anxiety, individuals with anxiety are more likely to have suicide risk (26). Previous studies have also found that anxiety symptoms can interfere with an individual’s daily activities and lead to suicidal ideation after 18 months (27), with such symptoms being common in the last week before suicide and associated with recorded increased suicide risk (28). Obsessive-compulsive symptoms, as highly correlated psychological issues with anxiety symptoms, have not yet reached a consensus in the industry regarding their relationship. Research suggests that anxiety is not a part of obsessive-compulsive symptoms, and obsessive-compulsive symptoms at baseline are related to anxiety symptoms two years later (29). Obsessive-compulsive symptoms cause distress, often manifested as anxiety (30), and more severe anxiety symptoms may exacerbate the impairment of patients’ quality of life (31, 32) and more functional impairment (33), thus anxiety symptoms may mediate the relationship between obsessive-compulsive symptoms and suicide risk.

### Role of sleep disturbances

Additionally, sleep disturbances, which are highly comorbid with depression and anxiety (34, 35), are also suicide risk factors that deserve our attention. Sleep disturbances can bring intense feelings of distress to individuals. According to the escape theory of suicide, the purpose of suicide is to avoid suffering (36). Therefore, sleep disturbances are likely to lead to suicide risk, which has been confirmed in many studies (37, 38). A cross-sectional study showed that compared to adolescents with longer sleep durations, those with shorter sleep durations have a higher likelihood of making suicide plans (39). The results of two meta-analyses indicate that sleep disturbances significantly increase suicide risk in both the general population (40) and in groups diagnosed with mental disorders (41). Furthermore, in psychological autopsy studies of completed suicides and studies of hospitalized groups who have attempted suicide, insomnia was found to be a proximal warning signal, appearing as early as one week before suicide (42–44). It is well-known that sleep disturbances are associated with many psychological disorders, as found in both cross-sectional and prospective studies (45, 46). Obsessive-compulsive symptoms, due to their recurring and distressing intrusive thoughts (47), may severely affect the sleep quality of adolescents (48). Clinical samples have also found that the incidence of delayed sleep–wake phase disorder in patients with obsessive-compulsive disorder is higher than in healthy control groups (49), thus sleep disturbances may mediate the relationship between obsessive-compulsive symptoms and suicide risk.

Additionally, obsessive-compulsive symptoms and suicide risk are significantly influenced by gender (7, 50). Females tend to develop obsessive-compulsive symptoms later than males (51), and there are differences in the incidence of various symptoms between the two (52). Females typically have an episodic course with stressful life events, while males often have a chronic course, with insidious onset and higher severity of symptoms (53, 54). Studies have also found that among individuals with obsessive-compulsive symptoms, females have significantly higher anxiety and depression scores than males (51). Similarly, in a sample of high school students, female patients with obsessive-compulsive disorder (OCD) reported significantly higher depression scores than their male counterparts (55). In the field of suicide research, gender differences in suicidal behavior, also known as the “suicide paradox,” were proposed as early as 1998 (56). Females often exhibit a higher rate of suicide attempts, but males have a higher likelihood of completed suicide. Additionally, a study involving 273 patients admitted due to suicide attempts revealed that females had a higher prevalence of anxiety symptoms (57) and females were more likely to exhibit traditional suicide risk factors such as depression (58). These findings suggest that in females, obsessive-compulsive symptoms are more likely to influence suicide risk through anxiety and depression. Therefore, there may be gender differences in the relationship between obsessive-compulsive symptoms and suicide risk. However, previous studies rarely explore gender differences within the context of the relationship between obsessive-compulsive symptoms and suicide risk.

In summary, this study aims to explore the mediating roles of depressive symptoms, anxiety symptoms, and sleep disturbances in the relationship between obsessive-compulsive symptoms and suicide risk, as well as differences at the gender level. Our hypotheses are as follows: (1) Obsessive-compulsive symptoms can positively predict suicide risk. (2) Depressive symptoms, anxiety symptoms, and sleep disturbances mediate the relationship between obsessive-compulsive symptoms and suicide risk.

## Methods

### Participants and procedure

Participants were recruited through convenience sampling from the outpatient department of a specialized psychiatric hospital following their initial consultation. Subsequently, they underwent a detailed assessment using standardized scales. Data collection and storage were conducted via mobile or computer terminals. Informed consent was obtained electronically or in writing from all participants and their legal guardians prior to the study.

Ultimately, the study enrolled a total of 526 participants, including 396 females (75.29%) and 130 males (24.71%), with a mean age of 15.39 years (SD = 1.23; age range 13–17 years). The study was approved by the research ethics committee of the institution where the first author is affiliated.

### Measures

Obsessive-compulsive symptoms were assessed using the obsessive-compulsive dimension of the Symptom Checklist-90 (SCL-90). This scale assesses the psychological state of patients over the past week, with the obsessive-compulsive dimension comprising 10 items (e.g., having to wash hands repeatedly, count numbers, or touch certain objects). A Likert five-point scale is used for scoring, ranging from 0 (not at all) to 4 (extremely). The reliability and validity of this scale have been validated in previous studies with adolescent populations (59), and in this study, the Cronbach’s *α* was 0.89.

Depressive symptoms were assessed using the Zung Self-Rating Depression Scale (SDS) (60)., which assesses the severity of depression in patients over the past week. This instrument consists of 20 items (e.g., “I feel down-hearted and blue,” “I have trouble sleeping at night”), with 10 items being reverse-scored (e.g., “I eat as much as usual,” “I look forward to the future with hope”), a Likert four-point scale is used for scoring, where 1 = none or very little of the time, 2 = sometimes or little of the time, 3 = often or a good part of the time, and 4 = always or most of the time. The reliability and validity of this scale have been validated in previous studies with adolescent populations (61), and in this study, the Cronbach’s *α* was 0.92.

Anxiety was measured using the Zung Self-Rating Anxiety Scale (62), which also assesses the severity of anxiety in patients over the past week. This tool consists of 20 items (e.g., “I feel more nervous and anxious than usual;” “I feel afraid for no reason”), with 5 items being reverse-scored (e.g., “I feel everything is fine and nothing unfortunate will happen;” “I feel calm and can sit quietly”), a Likert four-point scale is used for scoring, where 1 = none or very little of the time, 2 = sometimes or little of the time, 3 = often or a good part of the time, and 4 = always or most of the time. The reliability and validity of this scale have been validated in previous studies with adolescent populations (61), and in this study, the Cronbach’s *α* was 0.80.

Sleep disturbances were assessed using the sleep-related items from the Symptom Checklist-90 (SCL-90), which assess the sleep status of patients over the past week (e.g., difficulty falling asleep). A Likert five-point scale is used for scoring, ranging from 0 (not at all) to 4 (extremely severe). The reliability and validity of this scale have been validated in previous studies with adolescent populations (59), and in this study, the Cronbach’s *α* was 0.89.

Suicide risk was assessed using the Suicide Ideation Self-Rating Scale, which consists of 26 items divided into four subscales: Despair, Optimism, Sleep, and Dissimulation (e.g., “I often feel pessimistic and disappointed.”). The Optimism subscale is reverse-scored (e.g., “How wonderful it is to live in this rich and colorful era.”). In this study, the total scores of the Despair, Optimism, and Sleep subscales were included in the analysis. The reliability and validity of this scale have been validated in previous studies with adolescent populations (63), and in this study, the Cronbach’s *α* was 0.86.

### Statistical analyses

Data analysis was conducted using IBM SPSS version 26.0. Initially, Harman’s single-factor test was performed to assess common method bias. The results indicated that the variance explained by the first factor, both before and after rotation, was below the threshold of 40% (30.9%), suggesting that common method bias was not a significant concern in this study.

Subsequently, descriptive statistics and correlation analyses were performed to explore the preliminary relationships among the variables. Based on these analyses and controlling for gender and age, we used the Mplus 8.0 to examine the impact of obsessive-compulsive symptoms on suicide risk, as well as the mediating roles of anxiety symptoms, depressive symptoms, and sleep disturbances. Finally, a Bootstrap procedure with 5,000 resamples was employed to test the mediating effects.

Given that the questionnaire required completion of all items before submission, there were no missing values in the questionnaires used in this study.

## Results

### Descriptive statistics analysis

As shown in Table 1, gender is negatively correlated with age and positively correlated with obsessive-compulsive symptoms, depressive symptoms, anxiety symptoms, and suicide risk. Age is significantly negatively correlated with depressive symptoms and suicide risk. All other variables are significantly positively correlated with each other.

**Table 1:** Descriptive statistics and correlation analyses.

Variables	*M*	*SD*	1	2	3	4	5	6
1. Sex	-	-	-					
2. Age	15.39	1.23	−0.16***	-				
3. OCS	3.21	0.83	0.17***	0.01	-			
4. DS	55.94	12.01	0.14**	−0.09*	0.45***	-		
5. AS	47.63	11.22	0.18***	−0.08	0.50***	0.85***	-	
6. SD	9.40	4.07	0.05	0.04	0.36***	0.51***	0.56***	-
7. SR	15.43	4.69	0.27***	−0.09*	0.47***	0.52***	0.48***	0.37***

OCS, obsessive-compulsive symptoms; DS, depressive symptoms; AS, anxiety symptoms; SD, sleep disturbances; SR, suicide risk. *p < 0.05, **p < 0.01, and ***p < 0.001.

### Mediation analysis

Prior to testing the mediation effects, we first examined the direct effect of obsessive-compulsive symptoms on suicide risk after controlling for gender and age. The results of the path analysis indicated that obsessive-compulsive symptoms positively predict suicide risk. The model is a saturated model.

**Figure 1:**
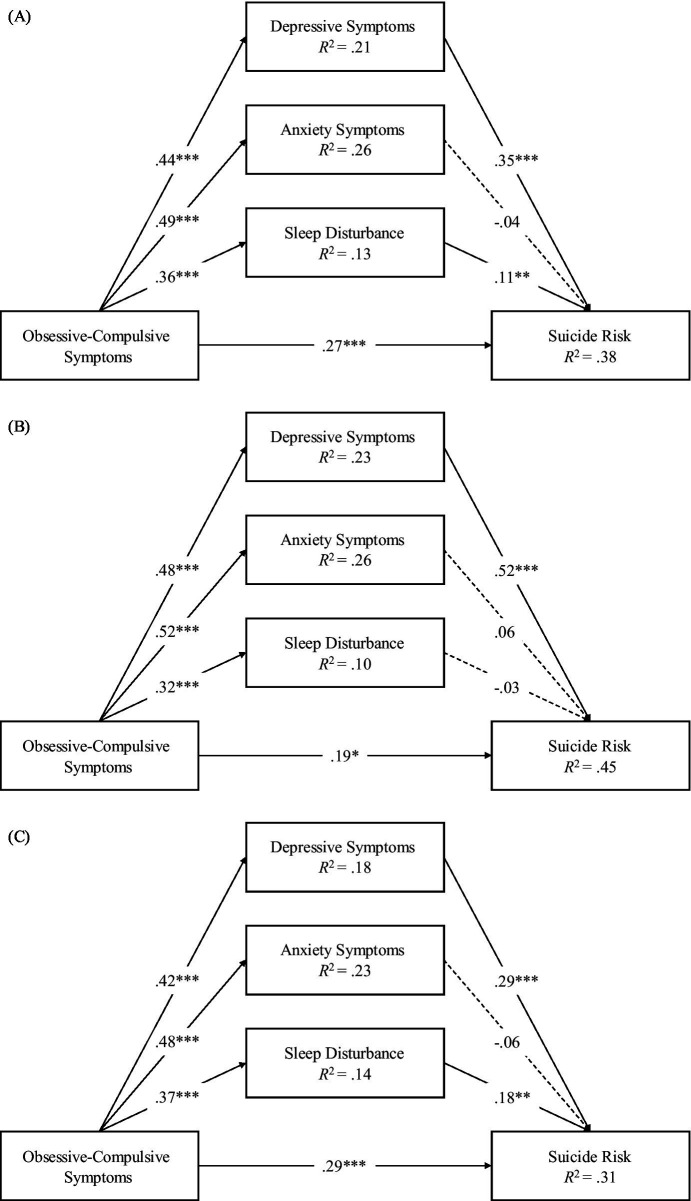
Mediation models. **(A)** The mediation model for the total sample, **(B)** the mediation model for males, **(C)** the mediation model for females. Standardized coefficients are shown. *p < 0.05, **p < 0.01, and ***p < 0.001.

In order to explore the underlying mechanisms between obsessive-compulsive symptoms and suicide risk, we incorporated depressive symptoms, anxiety symptoms, and sleep disturbances in the direct effect model simultaneously. The model is a saturated model, and the results are shown in Figure 1A. Path analysis shows that obsessive-compulsive symptoms positively predict depressive symptoms, anxiety symptoms, sleep disturbances, and suicide risk, depressive symptoms positively predict suicide risk, and sleep disturbances positively predict suicide risk.

To further explore the differences in underlying mechanisms between males and females, we constructed separate models for each gender group, as shown in Figures 1B,C. First, after constraining the main paths for males and females to be equal, the model fit showed significant changes (Δ*χ*^2^ = 22.69, Δdf = 7, *p* = 0.002), indicating that there were significant differences between the models for males and females. It was found that in the male group, obsessive-compulsive symptoms positively predicted depressive symptoms, anxiety symptoms, sleep disturbances, and suicide risk, and depressive symptoms positively predicted suicide risk. In the female group, obsessive-compulsive symptoms positively predicted depressive symptoms, anxiety symptoms, sleep disturbances, and suicide risk, depressive symptoms positively predicted suicide risk, and sleep disturbances positively predicted suicide risk.

The mediation effects were tested using the Bias-Corrected Bootstrap procedure. As show in Table 2, in the overall sample, Obsessive-compulsive symptoms can positively influence suicide risk through depressive symptoms and sleep disturbances. However, we did not find that obsessive-compulsive symptoms significantly influence suicide risk through anxiety symptoms.

**Table 2:** Path coefficients and mediation analyses.

Pathways	Total sample (*N* = 526)			Male (*n* = 130)			Female (*n* = 396)		
	*B* [95% CI]	*β*	*p*	*B* [95% CI]	*β*	*p*	*B* [95% CI]	*β*	*p*
Direct pathways									
OCS-DS	6.340 [5.229, 7.452]	0.439	< 0.001	6.365 [4.293, 8.438]	0.479	< 0.001	6.250 [4.929, 7.572]	0.419	< 0.001
OCS-AS	6.578 [5.571, 7.584]	0.488	< 0.001	6.368 [4.516, 7.923]	0.523	< 0.001	6.623 [5.421, 7.825]	0.475	< 0.001
OCS-SD	1.766 [1.371, 2.161]	0.361	< 0.001	1.650 [0.786, 2.376]	0.321	< 0.001	1.822 [1.377, 2.269]	0.373	< 0.001
OCS-SR	1.506 [1.061, 1.951]	0.267	< 0.001	1.244 [0.228, 2.097]	0.187	0.021	1.466 [0.993, 1.939]	0.291	< 0.001
DS-SR	0.137 [0.087, 0.187]	0.351	< 0.001	0.262 [0.140, 0.364]	0.523	< 0.001	0.097 [0.045, 0.148]	0.286	< 0.001
AS-SR	−0.169 [−0.073, 0.040]	−0.040	0.563	0.034 [−0.111, 0.155]	0.062	0.656	−0.022 [−0.080, 0.036]	−0.062	0.454
SD-SR	0.126 [0.031, 0.222]	0.110	0.010	−0.041 [−0.244, 0.163]	−0.031	0.704	0.181 [0.078, 0.283]	0.175	< 0.001
Mediation pathways									
OCS-DS-SR	0.869 [0.501, 1.335]	0.154	-	1.666 [0.684, 3.008]	0.251	-	0.604 [0.242, 1.001]	0.120	-
OCS-AS-SR	−0.111 [−0.460, 0.233]	−0.020	-	0.214 [−0.740, 1.251]	0.032	-	−0.148 [−0.538, 0.151]	−0.029	-
OCS-SD-SR	0.223 [0.034, 0.457]	0.040	-	−0.067 [−0.471, 0.329]	−0.010	-	0.329 [0.127, 0.541]	0.065	-

OCS, obsessive-compulsive symptoms; DS, depressive symptoms; AS, anxiety symptoms; SD, sleep disturbances; SR, suicide risk. 5,000-time bias-corrected confidence intervals are shown for mediation pathways.

In both male and female groups, Obsessive-compulsive symptoms can positively influence suicide risk through depressive symptoms. However, we did not find that obsessive-compulsive symptoms significantly influence suicide risk through anxiety symptoms. Among females, we observed that obsessive-compulsive symptoms can positively influence suicide risk through sleep disturbances, but the finding that was not replicated in the male group.

## Discussion

This study investigated the impact mechanisms of obsessive-compulsive symptoms on suicidal risk among psychiatric outpatient adolescents, as well as the mediating roles of depressive symptoms, anxiety symptoms, and sleep disturbances. The results indeed found the detrimental impact of depressive symptoms and sleep issues, with significant gender differences observed.

Initially, we constructed a direct effects model, and the results indicated that obsessive-compulsive symptoms are a positive predictor of suicidal risk in both male and female groups. This implies that the severity of an individual’s obsessive-compulsive symptoms is directly correlated with their risk of suicidality. This finding is consistent with our hypothesis and the results of previous studies (64). Intrusive obsessive thoughts that are unacceptable to the individual can induce intense feelings of shame and distress (65). Specifically, obsessive thoughts related to violence and horrific imagery (66) may provoke significant fear in the individual. Additionally, the substantial time spent on compulsive behaviors, coupled with the failure to control these behaviors, can lead to severe frustration. In an attempt to escape these distressing sensations, individuals may resort to suicidal ideation and behavior, thereby increasing their risk of suicidality.

Simultaneously, we found that obsessive-compulsive symptoms can positively predict suicide risk through depressive symptoms in both male and female groups. This is consistent with our hypothesis. Obsessive-compulsive symptoms may lead to depressive symptoms due to the impairment of an individual’s cognitive and social functioning. The frustration and distress caused by obsessive-compulsive symptoms and the failure to control compulsive behaviors may result in negative self-evaluation and hopelessness—the core personal intrinsic features of depression (67–69). The perceived inability to change one’s circumstances may lead to a sense of entrapment, thereby increasing the risk of suicide (70), which partially validates the IMV model.

Additionally, we found that in both groups, obsessive-compulsive symptoms did not affect suicide risk through anxiety symptoms, which is inconsistent with our hypothesis. It might be due to the high comorbidity between anxiety symptoms and depressive symptoms (71), where the role of anxiety symptoms is overshadowed by depressive symptoms. According to the tripartite model of anxiety and depression (72), negative affect is a shared component of both anxiety and depressive symptoms. The failure to control obsessive behaviors and the presence of intrusive obsessive thoughts often lead to negative emotions such as pessimism, self-blame, and hopelessness. These negative emotions are the core of depressive symptoms, and thus depressive symptoms may dominate in this context. Similar results have been confirmed in previous studies (73).

Interestingly, we found that in females, obsessive-compulsive symptoms can positively predict suicidal risk through sleep disturbances, whereas the mediating role of sleep disturbances was not significant in males. When confronted with the stress and distress brought by obsessive-compulsive symptoms, males are more likely to employ externalizing behaviors such as impulsivity and aggression to cope, while females tend to utilize internalizing behaviors like rumination and worry (74). Consequently, females are more prone to experiencing sleep disturbances (75). Additionally, females experience more stressors during adolescence compared to males (76), and they exhibit more stress responses at equivalent levels of stress (77). These factors may contribute to females being more susceptible to sleep disturbances. Moreover, previous research has indicated that dysfunctional sleep beliefs are only associated with an increased risk of suicide attempts in females (78), and the direct impact of sleep deprivation on suicide is 2.5 times greater in females than in males (79).

Additionally, this study has several limitations. All data in this study were derived from participants’ self-reports, which may be subject to bias, Future research could incorporate multimodal assessments (such as clinical interviews and ecological momentary assessment) to reduce subjective bias and include objective measures (such as actigraphy for sleep monitoring). Moreover, this study was a cross-sectional study and thus unable to verify the direction of causality or dynamic changes between variables. For instance, the association between obsessive-compulsive symptoms and mediating variables may be influenced by reverse causality (e.g., individuals at high risk of suicide risks may exaggerate their obsessive-compulsive symptoms). Furthermore, mediation analyses using cross-sectional data (e.g., via the Bootstrap method) can only provide statistical evidence of indirect effects and cannot confirm the actual temporal sequence or causal chain (80). Future studies may employ longitudinal designs, for example, by tracking the temporal sequence of obsessive-compulsive symptoms → mediating variables → suicide risk at three time points to validate the causal chain. Alternatively, cross-lagged panel models (CLPM) or randomized controlled trials (RCTs) could be used to clarify the causal relationships between variables. In addition, the present study did not differentiate between clinical and non-clinical samples, which may have confounded the effects of symptom severity. Future research could compare individuals with clinical diagnoses with community samples to clarify the moderating role of symptom severity. And our participants were individuals seeking treatment in the psychiatric outpatient department, so caution should be exercised when generalizing our results to other populations. Despite these limitations, this study still holds significant theoretical and practical value. The results of this study enrich our understanding of suicidal risk and partially validate the Interpersonal-Psychological Theory of Suicide (IMV) model and its broad applicability. Clinically, it can provide more targeted objectives for suicide interventions among adolescents attending psychiatric outpatient clinics. Physicians should pay closer attention to adolescents presenting with obsessive-compulsive symptoms and depressive symptoms, as well as female adolescents with sleep disturbances, during consultations to prevent them from experiencing higher suicidal risks.

## Data Availability

The raw data supporting the conclusions of this article will be made available by the authors, without undue reservation.
